# Lipids in Psychiatric Disorders: Functional and Potential Diagnostic Role as Blood Biomarkers

**DOI:** 10.3390/metabo14020080

**Published:** 2024-01-23

**Authors:** Yana Zorkina, Valeria Ushakova, Aleksandra Ochneva, Anna Tsurina, Olga Abramova, Valeria Savenkova, Anna Goncharova, Irina Alekseenko, Irina Morozova, Daria Riabinina, Georgy Kostyuk, Anna Morozova

**Affiliations:** 1 Mental-Health Clinic No. 1 Named after N.A. Alekseev, Zagorodnoe Highway 2, 115191 Moscow, Russia; yshakova.v@serbsky.ru (V.U.); ochneva.a@serbsky.ru (A.O.); anna.tsurina@mail.ru (A.T.); abramova.ol@serbsky.ru (O.A.); savva9806@yandex.ru (V.S.); schelkanovaio@zdrav.mos.ru (I.M.); ryabininada1@zdrav.mos.ru (D.R.); kostyukgp@zdrav.mos.ru (G.K.); morozova.a@serbsky.ru (A.M.); 2 Department of Basic and Applied Neurobiology, V. Serbsky Federal Medical Research Centre of Psychiatry and Narcology, Kropotkinsky per. 23, 119034 Moscow, Russia; 3 Moscow Center for Healthcare Innovations, 123473 Moscow, Russia; goncharovaas4@zdrav.mos.ru; 4 Shemyakin-Ovchinnikov Institute of Bioorganic Chemistry, Russian Academi of Science, 142290 Moscow, Russia; 5 Russia Institute of Molecular Genetics of National Research Centre “Kurchatov Institute”, 2, Kurchatov Square, 123182 Moscow, Russia

**Keywords:** lipids, MDD, schizophrenia, bipolar disorder, anxiety disorder

## Abstract

Lipids are a crucial component of the human brain, serving important structural and functional roles. They are involved in cell function, myelination of neuronal projections, neurotransmission, neural plasticity, energy metabolism, and neuroinflammation. Despite their significance, the role of lipids in the development of mental disorders has not been well understood. This review focused on the potential use of lipids as blood biomarkers for common mental illnesses, such as major depressive disorder, anxiety disorders, bipolar disorder, and schizophrenia. This review also discussed the impact of commonly used psychiatric medications, such as neuroleptics and antidepressants, on lipid metabolism. The obtained data suggested that lipid biomarkers could be useful for diagnosing psychiatric diseases, but further research is needed to better understand the associations between blood lipids and mental disorders and to identify specific biomarker combinations for each disease.

## 1. Introduction

The most common mental illnesses are affective disorders and schizophrenia. Thus, among them are major depressive disorder (MDD), anxiety disorders (ADs), bipolar disorder (BPD), and schizophrenia (SCZ) [1]. These illnesses result in long-term disability and cause invalidity. Their disease courses have been characterized by emotional and cognitive disturbances, mood disorders, impaired functioning, and social isolation [1]. In recent years, advances in technology have allowed for the identification of many biological markers of mental illnesses, such as genomic, epigenomic, metabolic, and proteomic markers. However, much less attention has been paid to the lipid markers.

**Figure 1:**
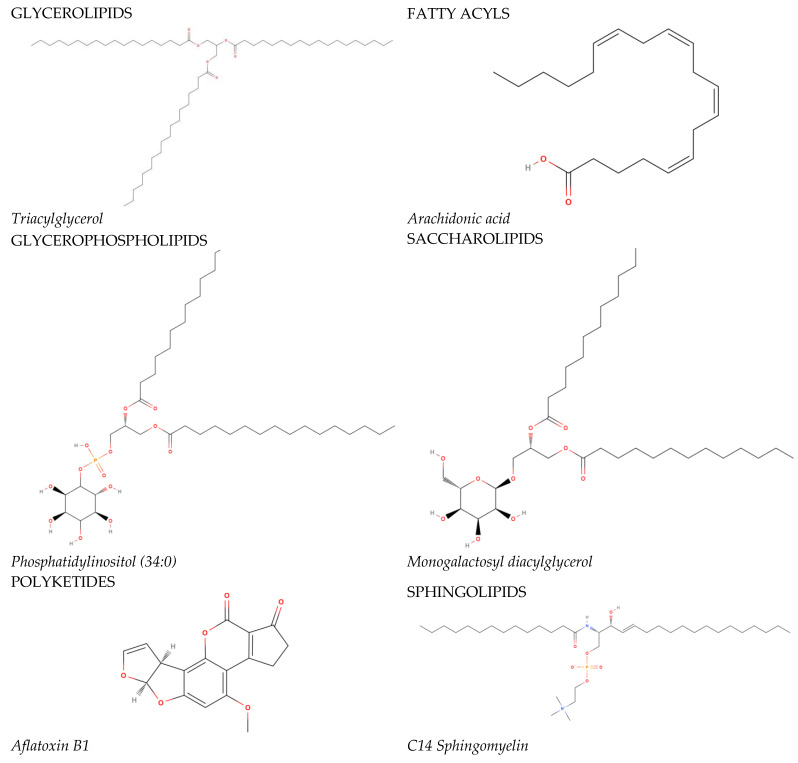

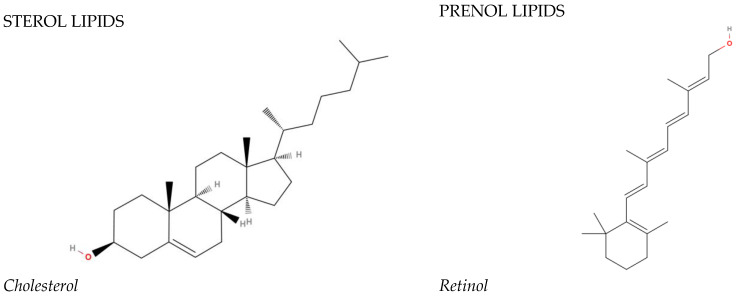
The main lipid groups and the examples of their representatives. The illustrations were prepared using the following program: https://molview.org/ accessed on 18 December 2023).

In 2005, the International Committee on the Classification and Nomenclature of Lipids identified eight classes of lipids; they have been displayed in the LIPID MAPS Structure Database [2]. Two fundamental ‘building blocks’ (ketoacyl groups and isoprene groups) form the basis of the LIPID MAPS classification system. Therefore, lipids are defined as hydrophobic or amphipathic small molecules that can arise, in whole or in part, from two types of condensation: based on the carbanions of ketoacyl thioethers and/or based on the carbocations of isoprene units. This classification system segregates eight categories of lipids: fatty acyls, glycerolipids, glycerophospholipids, sphingolipids, saccharolipids, polyketides (derived from the condensation of ketoacyl subunits), sterol lipids, and prenol lipids (derived from the condensation of isoprene subunits). The classification of these lipids is shown in Figure 1.

The brain is the organ that is enriched in lipids. Only adipose tissue contains a larger amount of lipids compared to brain tissue [3]. The entire variety of brain lipids is involved in a range of essential processes, the disruption of which can cause significant damage to the central nervous system (CNS).

Lipids are the structural components of cell membranes, which are involved in a set of processes in the cell, such as myelination, neurotransmission, synaptic plasticity, energy, metabolic processes, and inflammatory processes. Interventions in these processes may influence the development of psychiatric disorders and contribute to their pathogenesis [4].

The state of the cell membrane is extremely important for the functions of neurons and glial cells. In nerve cells, lipids comprise 50–60% of cell membrane components [5]. Lipids form a phospholipid bilayer, the basic structural unit of the membrane, which participates in the regulation of permeability. The three major classes of membrane lipids are glycerophospholipids (e.g., phosphatidylcholine (PC), phosphatidylserine (PS), phosphatidylethanolamine (PE), and phosphatidylinositol (PI)), cholesterol, and sphingolipids. The outer layer of the plasma membrane mainly consists of PC and sphingomyelin (SM); PE and PS represent the predominant phospholipids of the inner layer. PI is also localized in the inner part of the membrane and plays an important role in cell signaling [5]. Sphingolipids (SPs) contain long-chain fatty acids, which provide inter-lipid associations in the lipid bilayer [6]. Cholesterol acts as a “strengthening link” in the structures of membranes, providing them with the necessary strength and stability. Cholesterol affects membrane fluidity and increases the level of friction between membrane flaps [7]. The relative size and degree of fatty acid saturation in lipids affect membrane curvature, fluidity, and thickness [8]. Moreover, lipids can modulate the activity of membrane proteins with lipid-binding domains by recruiting them to specific membrane compartments or subdomains [9]. Lipids also participate in intracellular signaling, where they act as secondary messengers. The most common are diacylglycerol (DG) and inositol triphosphate (IP3) [10]. 

Myelination plays a crucial role in signal transduction and the proper functioning of the CNS. The main function of myelin is to provide electrical insulation of axons for sufficiently efficient transmission of action potentials. Myelin is characterized by an extremely high lipid content (~80% of dry weight) and a peculiar lipid composition in which the ratio of cholesterol to phospholipids (mainly ethanolamine phosphatide and phosphatidylcholine) to glycolipids (e.g., galactosylceramide and sulfatide) is approximately 2:2:1. Specific SPs and glycerides are substances that cover nerve fibers and accelerate the transmission of nerve impulses [11]. Myelin is particularly high in saturated and monounsaturated lower and higher fatty acids. Phospholipids containing such fatty acids may contribute to the electrical insulation of axons by reducing their membrane fluidity [12]. 

The functioning and activity of cellular receptors depend on the interactions between the proteins and lipids that comprise the bilipid layer of the cell membrane [13]. Firstly, lipids are involved in the regulation of synapse development and plasticity. For example, levels of tropomyosin receptor kinase B, a crucial protein in synapse development, are regulated via cholesterol levels [14]. Secondly, lipids participate in the release of presynaptic vesicles [15]. Third, lipids regulate neurotransmitter receptors independently, mostly through direct interactions. For example, cholesterol has been shown to function as a direct allosteric regulator of G protein-coupled receptors [16]. Abnormal phospholipid changes have been reported to disturb the functions of ion channels, neurotransmitters, and cell signaling [17].

Lipids also control neuroplasticity. Glycerophospholipids (GPs) and phosphoinositides are important regulators of dendritic spine plasticity. Lipids also influence dendritic spine plasticity by covalently binding to key synaptic proteins via palmitoylation, which can reversibly modulate protein function [18]. Neutral sphingomyelinases also regulate synaptic potentiation. Previous studies have demonstrated at least two different functions of lipids in plasticity processes: altering the functions of synaptic proteins through the palmitoylation mechanism and linking cytoskeletal regulators to membrane remodeling [19]. SPs modulate structural plasticity and neuronal dynamics through lipid–cytoskeletal interactions [20]. Neuronal activity can induce rapid changes in lipid metabolism. It rapidly modulates GP and SP levels. Several studies have shown the effects of ceramide (Cer) metabolism on neuronal susceptibility to death and plasticity process [21]. Cholesterol-deficient neuronal cells exhibit reduced synaptic transmission and impaired synaptic plasticity [4].

Brain tissue needs a large amount of energy. Neurons in the adult brain mainly depend on glucose as an energy source. However, about 20% of the total energy requirements of the adult brain are provided through the oxidation of fatty acids. It has been considered that fatty acid oxidation occurs almost exclusively in astrocytes, and carnitine and fatty acids can be transported from the blood to the astrocytes [22]. Brain mitochondria are characterized by a number of special features. The lipid-to-protein ratio of phospholipids, or cholesterol, is lower in brain mitochondria compared to other organelles [23]. In particular, mitochondria do not contain SM and glycosphingolipids. The major phospholipids of mitochondrial membranes are PC and PE, mainly located in the inner membrane. PI and PS are almost equally distributed on both membranes [23].

Inflammatory processes have been observed during many mental illnesses [24]. Microglial cells are activated during inflammation and perform phagocytosis to counteract inflammation [25]. Once abnormalities are detected, complex remodeling of the lipid composition of microglial cells occurs, providing inflammatory signaling and effector functions [26]. Microglial cells contain receptors for low-density lipoproteins (LDLs) that regulate inflammatory signaling [27]. Cers also promote inflammation and microglia activation [21]. Several lipids represent the sources of pro-inflammatory cytokines that contribute to pathologic neuroinflammatory processes. For example, under certain conditions, arachidonic acid (AA) can produce pro-inflammatory mediators, such as prostaglandins (PGs) and leukotrienes [28]. Long-chain PUFAs (polyunsaturated fatty acids), representing a source of eicosanoids and docosanoids, play an important role in neuroprotective and anti-inflammatory effects in the CNS [4]. SMs also participate in neuroinflammation through cytokine release, microglia activation, and other immune processes [29].

**Figure 2:**
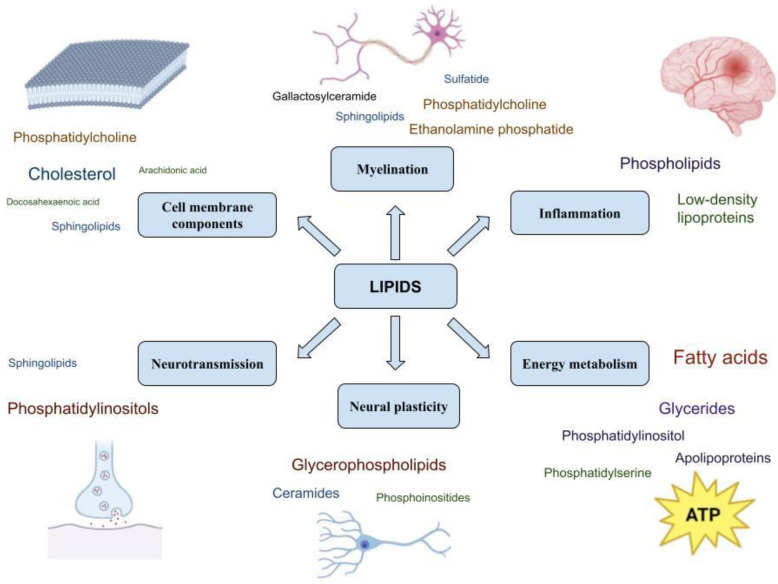
Lipids and their functions in the nervous system.

The role of lipids in physiological processes important for the functioning of the nervous system is summarized in Figure 2.

Therefore, lipids play a major role in the functioning of the nervous system, and alterations in lipid metabolism may influence mental disorder development. 

Changes in lipid metabolism in brain tissue may contribute to the pathogenesis of neuropsychiatric diseases. The presence of the blood–brain barrier prevents the free penetration of compounds into the brain. Small lipophilic molecules can pass into brain tissue via passive diffusion; at the same time, all other lipids enter the brain via transcytosis or special transport proteins [23]. For example, unbound long-chain fatty acids can diffuse through the membranes. Meanwhile, cholesterol is almost entirely synthesized in the brain, so its concentration in the blood cannot reflect the processes occurring in the central nervous system. It has been hypothesized that fatty acid metabolism in specific regions of the hypothalamus functions as sensors of nutrient availability that are involved in integrating energy balance through the control of multiple nutritional and hormonal signals. In other brain regions, no differences in glucose and fatty acid metabolism were found, depending on nutritional status [23]. The study of brain lipid composition is an important and urgent task from the point of view of fundamental science. However, for practical purposes, it is necessary to study its associations with blood lipid content. In this review, we have chosen to specifically focus on blood biomarkers in order to determine the potential of using lipids in the potential diagnostics of mental illness. Our review summarized recent data on the associations of lipid blood composition with mental disorders such as SCZ, BPD, MDD, and AD. The first part of this review addressed the effects of changes in blood lipid composition on mental illness, while the second part of this review discussed the effects of drugs used in the treatment of mental disorders on lipid metabolism.

## 2. Lipids and Their Role in Neuropsychiatric Disorders

Lipids influence several pathophysiologic pathways that are involved in the development of psychiatric illnesses [30]. The most pronounced effect in the literature has been shown for SCZ and MDD.

Disruption of lipid function is one of the components of SCZ pathogenesis [31]. Yao et al. demonstrated a direct link between abnormal phospholipid levels and disrupted neurochemical parameters, such as SCZ-associated abnormal dopamine and glutamate levels [32]. Phospholipid metabolism abnormalities occur during the progression of SCZ. Most notably, phospholipase A2 (PLA2) activity increases and the level of PUFA integration into phospholipids decreases [31]. The association between PLA2 activity and the dopamine system has also been demonstrated [33]. In particular, it was demonstrated that PUFA dissociation and saturated fatty acid (SFA) incorporation in membrane phospholipids are enhanced in SCZ patients. Decreased levels of membrane phospholipid precursors in the brains of SCZ patients indicate reduced synthesis of PC and PE. Abnormal expression of enzymes and impaired homeostasis of membrane lipids in patients have been associated with the imbalance of phospholipid breakdown and remodeling under the influence of increased oxidative stress. Phospholipid metabolism plays a critical role in the process of synaptic growth, and its dysfunction has been associated with abnormal neuronal development in SCZ [17]. SM and Cers also exert an effect on the presynaptic release of dopamine [34].

In the field of depression research, Andreas Walther et al. proposed their model of lipid involvement in the pathogenesis of depression [35]. This model is based on the chronic stress effects. Chronic stress has been thought to trigger two main pathways: the hypothalamic–pituitary–adrenal axis (HPA) and neuroinflammation [35]. 

Chronic stress leads to HPA hyperactivity. Elevated glucocorticoid levels increase phospholipase D activity [35]. Increased phospholipase D activity enhances the conversion of PC and PE into phosphatidic acid, as well as lysophosphatidylcholine (LPC) and lysophosphatidylethanolamine (LPE). Due to its chemical properties, phosphatidic acid is rapidly converted into DG. DG, LPC, and LPE cause membrane buckling and destabilization, allowing for a greater glucocorticoid influx into the cell. Together with the above mechanism, elevated glucocorticoid levels decrease triacylglycerol hydrolase expression and enhance triacylglycerol (TAG) biosynthesis by increasing the level of diacylglycerolacyltransferase 2. Decreased triacylglycerol hydrolase expression and increased TAG biosynthesis raise the level of TAG [35]. TAG, in turn, is associated with increased glucocorticoid levels.

Chronic stress also leads to the dysregulation of inflammation. Excess pro-inflammatory cytokines and phasic reagents increase the level of PLA2 [35]. Increased PLA2 activity induces the conversion of PC-containing linoleic acid into AA. AA is subsequently converted into PGs, including pro-inflammatory cytokines (e.g., PGA2, PGD2, PGE2, PGF2, PGH2, and PGI2) [35]. PGs further enhance inflammatory responses. 

Increased saturated fatty acid-rich phospholipids, namely lysoPS (16:0), lysoPS (18:0), and SM (24:0), have been associated with inflammation and oxidative stress responses in depressed patients [35]. The elevation of δ-6 desaturase activity in patients with depressive symptoms has been demonstrated. δ-6 desaturase converts linoleic acid into AA, which is a precursor of pro-inflammatory products [36].

Several studies have suggested that omega-3 fatty acid deficiency can decrease dopamine levels, D2 receptor expression and mRNA, presynaptic dopamine vesicle amount, and increase dopamine cleavage [37]. Moreover, its deficiency also downregulates tyrosine hydroxylase activity, which results in reduced dopamine levels and depressive symptomatology development [38].

Increased Cer concentrations may also contribute to the progression of depression, as Cers may affect dopamine transporter function by decreasing dopamine transport and increasing 5HT transport [39]. Moreover, it has been reported that increased Cers may affect monoamine neurotransmitter reuptake and initiate a biological cascade that leads to the downregulation of serotoninergic neurotransmission, which represents another pathophysiologic hallmark of depression [38].

Most of the changes accompanying psychiatric disorders primarily affect brain structures. Lipid metabolism impairments during mental diseases also primarily occur in the CNS tissues. Nevertheless, it is necessary to use the available research methods for diagnostic purposes in clinical practice. In this regard, the second part of this review is devoted to the study of blood lipid composition in mental disorders, as these data may have diagnostic value. Although the role of lipids in the pathophysiological pathways of mental illnesses have not been sufficiently investigated and mainly concern the mechanisms of schizophrenia and depression, a significant number of studies have analyzed lipids as potential biomarkers of psychiatric diseases.

Changes in lipid profiles have been consistently observed in the blood serum and plasma across patients with psychiatric diagnoses. 

In particular, a lot of studies have focused on identifying reliable blood lipid indicators in SCZ. In total, over 29 studies have assessed lipid changes in the blood of patients with SCZ compared to healthy controls (Table S1). Two studies were conducted without a control group (Table S1). Most of the observed studies included patients that received treatment, but three of them also included patients with first-episode psychosis (FEP) (Table S1). However, only nine of the described studies involved more than 100 individuals (Table S1). 

Nine studies evaluated the broad panels of different lipid markers in patients with SCZ [31,34,40,41,42,43,44,45,46] and detected statistical differences in the following lipid classes: fatty acyls, sterols, glycerolipids, sphingolipids, glycerophospholipids, and products of lipid metabolism. Several studies included information about lipids associated with the membranes of erythrocytes [31,47,48]. The most consistent data from the reviewed studies were obtained for PC, PE, SM, and triacylglycerols (TGs). In particular, reduction in these lipid species was mostly demonstrated. Malondialdehyde—the marker of oxidative stress—was also increased in all the concerned studies. Moreover, a meta-analysis considering malondialdehyde in SCZ was conducted [49]. It was shown that medically treated SCZ patients were more affected by the increased oxidative stress, but malondialdehyde levels were elevated in both the treated and untreated groups, in contrast to other markers of oxidative stress. Bile acids were investigated in two studies, and their levels decreased in both of them [40,50]. Calcifediol was reduced in one observed study [50]. High-density lipoprotein (HDL) was decreased in two studies [51,52]. Studies considering fatty acyl and Cer levels received inconsistent results: some lipid types were decreased and some were increased. The levels of PC and PE did not differ significantly in FEP patients and medicated patients [53,54]. The data addressed to PUFA concentrations were found to be inconsistent. 

It is worth highlighting those studies that do not identify differences between healthy individuals and patients with SCZ but rather make associations between blood lipids and symptoms of the illness. In some studies, changes in lipid levels in SCZ patients have been associated with Positive and Negative Syndrome Scale (PANSS) scores. For example, such correlations have been detected for shorter-chain TGs [41] and oxysterols [55]. Nandeesha (2023) [52] showed that total cholesterol (TC) and TG levels were negatively correlated with cognitive scores. Plasma calcifediol levels and the ratio of cholestanol to tchol were found to be negatively correlated with Montreal Cognitive Assessment (MOCA) scores [55]. Baseline membrane linoleic acid levels in SCZ with ultra-high risk (UHR) were associated with conversion to psychosis. Sterol, fatty acid, and phospholipid membrane compositions improved the prediction of the psychosis onset [47]. TC levels were positively associated with the Repeated Battery for the Assessment of Neuropsychological Status (RBANS) subscale scores of immediate memory and language [56]. These results further suggest the potential use of blood lipid profiles for the assessment of SCZ symptomatology.

For MDD, we described nineteen studies (Table S1), which included one study considering postpartum depression [57] and four studies considering depression symptoms in the healthy population (Table S1). Four studies above them were conducted on drug-naïve patients (Table S1). Only four studies on MDD patients included more than one hundred individuals (Table S1). Six studies evaluated a broad panel of different lipid markers [34,58,59,60,61,62]. Consensual data were received for LPC and LPE measurements, which were increased in the observed studies. PC and malondialdehyde levels were also mostly elevated in the described studies. On the contrary, acylcarnitine (CAR), calcifediol, SM, and bile acids were mostly decreased in the reviewed studies. Inconsistent data were received for PE, PI, Cers, TGs, PUFAs, and SFAs. Two studies on MDD patients and one on postpartum depression individuals reported a reduction in HDL levels. One study indicated an increased level of LDL. Researchers have investigated the levels of cholesteryl ester (CE), TC, sterols, and calcifediol in healthy people with depressive symptoms. Associations with mental symptoms were shown for TC and sterol lipids in women. Several studies have indicated an association between the lipid concentrations of octadecyl-phosphatidylethanolamine (PE-O) [58], SM, and PC-O [63] and symptom severity according to the specific scales. 

Fourteen studies were dedicated to the investigation of lipid changes in BPD, including two conducted on drug-naïve patients (Table S1). Compared to the studies on SCZ and MDD, fewer studies were conducted on patients with BPD. Only one study evaluated the associations of CAR, CE, calcifediol, PE, PC, LPC, LPE, PS, and SM with disease symptoms (Table S1). Cer and PI levels were increased in two and three studies, respectively. PUFA, TC, and TG changes showed inconsistent associations. 

We described 24 studies devoted to the evaluation of blood lipid biomarkers in AD (Table S1). Eleven studies considered blood lipid constitution during general anxiety disorder (GAD), or AD, including one performed on pregnant women (Table S1). One study described post-stroke anxiety [64]. Some studies have focused on comorbid psychiatric pathology, such as comorbid AD and MDD or comorbid AD and Parkinson’s disease. The other six works included population studies, which investigated blood lipid biomarkers in individuals with anxiety symptoms (Table S1). The majority of this research included healthy control or other comparison groups with mental disorders, and only eight did not. Six studies have been conducted on a broad sample of individuals (more than 300) (Table S1). Nevertheless, only a few large metabolomic studies assessing lipid blood constitution in AD were performed [63,65,66]. These broad metabolomic studies have revealed changes in a number of lipids of various classes: fatty acyls, sterol lipids, GP, SP, and glycerolipids [63,65,66].

Most of these studies have focused on investigating the changes in lipoproteins, TGs, and cholesterol. Regarding lipoprotein levels, the results were questionable and multidirectional. Nevertheless, in almost all of the papers that were reviewed, anxiety symptomatology was accompanied by an increase in TGs [67,68,69,70,71] and, in only one, by a reduction [65]. For cholesterol, the reviewed results were also found to be inconsistent. A number of studies have investigated the change in PUFAs in blood during anxiety states and mainly demonstrated their decrease [65,72,73]. Regarding the SFA elevation, multidirectional results have been shown. One study identified a decreased carnitine (propionylcarnitine) level [66]. Bile acid changes were also shown in one study reflecting anxiety symptomatology in MDD [74]. Alterations in Cers have also been found in comorbid pathologies. In particular, Xing et al. demonstrated a positive association of Cer C 20:0 levels with anxiety symptoms in Parkinson’s disease [75]. Unidirectional changes were detected when studying the levels of 20-oxo-22,23,24,25,26,27-hexanorvitamin D3 and malondialdehyde. Thus, decreased levels of 20-oxo-22,23,24,25,26,26,27-hexanorvitamin D3 accompanied anxiety symptomatology [76,77]. Malondialdehyde, on the contrary, was increased in AD patients [62,77]. No changes were identified for calcifediol [78].

Among the addressed studies, one was dedicated to the transdiagnostic lipid markers between four illnesses: MDD, BPD, AD, and SCZ [79]. The authors tried to indicate these transdiagnostic lipid subtypes. Researchers have suggested that 10 lipids can be used for diagnostics across psychiatric disorders. Along with these lipid types, the marker of oxidative stress, malondialdehyde, was increased in all the mentioned psychiatric disorders according to the observed studies. The levels of CAR and SM were also decreased in all reviewed psychiatric disorders. Thus, a number of lipid biomarkers were altered in these mental illnesses. The unidirectionality of some of these changes may indicate the diagnostic potential of blood lipid estimation.

Taken together, these studies highlight the need for systematic analysis of the robustness of observed lipidome alterations and their specificity to a single disorder. Future studies would also need to consider the comorbidities commonly linked to psychiatric disorders. The summarized information on lipids as diagnostic biomarkers is presented in Table 1. The description of all the reviewed studies is presented in Table S1.

## 3. Effect of Medications on Lipid Metabolism

Among the possible confounding factors affecting lipidome measurements, psychopharmacologic treatment effects have been particularly well documented. In this section, two classes of psychotropic drugs will be considered: neuroleptics and antidepressants.

### 3.1. Antipsychotics

Treatment with antipsychotic drugs has been associated with metabolic disturbances, and side effects of these drugs, such as obesity, hypertriglyceridemia, and glucose dysregulation, have been linked to these processes [129]. Among others, neuroleptic therapy affects lipid homeostasis [130,131]. The large amount of data concerns cholesterol and lipoprotein changes. In particular, it was shown that typical and atypical antipsychotics increase the levels of cholesterol, TG, and LDL. Antipsychotic drugs produce metabolic side effects with a different extent depending on their type. For example, olanzapine and clozapine demonstrated the worst side effect profiles, while the most favorable profiles have been shown for aripiprazole, brexiprazole, cariprazine, lurasidone, and ziprasidone [131]. The negative effect of olanzapine on lipid homeostasis has been detected even in patients with a first episode of psychosis [132]. Comparative research revealed that haloperidol and quetiapine can increase lipid levels; ziprasidone probably improves lipid levels, while risperidone can produce both effects [133]. Both atypical and typical antipsychotics may worsen lipid peroxidation [49]. However, the potential mechanisms of dyslipidemia induced by antipsychotics are of the greatest interest.

Neuroleptics inhibit cholesterol biosynthesis in vitro by reducing the activities of the enzymes involved in this pathway, leading to the accumulation of various sterol intermediates [134,135]. In vitro, clozapine was found to be the most prominent stimulator of fatty acid, TG, and phospholipid biosynthesis. Antipsychotic drugs induce the inhibition of cholesterol biosynthesis, affecting the same enzymes with a different relative activity: ziprasidone > haloperidol > risperidone [129]. Inhibition of its biosynthesis leads to impaired hormone signaling for insulin and somatostatin in vitro [135]. In addition, antipsychotics disrupt intracellular cholesterol trafficking by inhibiting cholesterol efflux from endolysosomes, thereby reducing the transport of endocytosed LDL cholesterol into the endoplasmic reticulum and Golgi apparatus [136]. As cationic amphiphilic drugs, antipsychotics alkalinize lysosomes, affecting lysosomal function, as has been shown with haloperidol [134]. In addition, antipsychotics increase LDL receptor transcription, thereby stimulating LDL endocytosis and exacerbating the intracellular accumulation of LDL-derived lipids [136].

Chlorpromazine and the antidepressant imipramine can increase cholesterol content in lysosomes and disrupt sterol regulatory element-binding protein (SREBP) mediated by the cholesterol-sensing system in the endoplasmic reticulum. Antipsychotic drugs induce the transcriptional activation of cholesterol and FA biosynthesis genes under the control of the transcription factors SREBP1 and SREBP2 [129]. It was shown that treatment with clozapine or risperidone enhanced lipogenesis and cholesterogenesis via the inhibition of PGRMC1/INSIG-2 and activation of SCAP/SREBP expression in rats. However, similar metabolic disturbances were not observed in rats treated with aripiprazole or haloperidol. Moreover, additional treatment with mifepristone effectively reversed the lipid abnormalities induced by atypical antipsychotics [137]. Thus, neuroleptics increase lipogenesis, decrease lipolysis, and enhance the antilipolytic effect of insulin in adipocytes. As a result, this leads to lipid accumulation in adipocytes [133].

Antipsychotics can alter membrane compartmentalization, which may differentially modulate the signaling cascade of the dopamine D2 receptor [138]. Antipsychotics demonstrate a higher affinity for SM compared to phosphatidylcholine. Cholesterol increased the affinity of these drugs to the lipid bilayer and resulted in the following ranking of neuroleptics by this factor and corresponding structural changes: risperidone >9-OH- risperidone> haloperidol. Studies performed on single lipids and mixtures consisting of lipids of biological origin demonstrated that antipsychotics can also modify D2 receptor activity by altering the lipid environment of the receptor [138].

The disruption of other lipid species during antipsychotic treatment has also been shown in human studies. SCZ patients treated with risperidone showed decreased levels of dihydroceramide, very long-chain Cers, and lysoPC in mononuclear cells [136]. Phosphorous magnetic resonance spectroscopy (2D chemical shift imaging (CSI)) allows for the study of membrane phospholipids and high-energy phosphates in vivo. Using this technique, the authors showed that risperidone stimulates the remodeling of neuronal and synaptic phospholipids. This drug increased the level of adenosine triphosphate in the left dorsolateral prefrontal cortex, left anterior temporal cortex, left insular cortex, basal ganglia, and anterior cerebellum, and increased the levels of phosphomonoesters, phosphodiesters, and phosphocreatine in these brain regions [139].

Neuroleptics, at doses recommended for the treatment of acute episodes of SCZ, can also cause distinct changes in the plasma levels of lipid peroxidation products. For example, quetiapine, which is also used in the treatment of depressive disorders, demonstrates the strongest antioxidant properties, in contrast to the pro-oxidant effects of risperidone, ziprasidone, haloperidol, and clozapine at low doses [140].

Almeida [141] investigated the lipidome changes in their blood plasma samples before and after 6 weeks of treatment with either risperidone, olanzapine, or quetiapine. Risperidone affected DG, ceramide 1-phosphates, TG, SM, and ceramide phosphoinositols. Olanzapine mainly affected the PS, PC, glycerophosphatidic acid PA, and glycerophosphoglycerol PG lipid classes. Quetiapine affected the lipid profiles of patients to a smaller extent. After medication with risperidone or olanzapine, the levels of LysoPC, PC, PE, C16 sphinganine, and adrenic acid were significantly increased, while the levels of linoleic acid, oleic acid, palmitoleic acid, γ-linolenic acid, and oxoglutaric acid were significantly decreased [50].

### 3.2. Antidepressants

Along with antipsychotic drugs, antidepressants also cause changes in blood lipid composition. Many studies have shown the negative effect of antidepressants on lipid metabolism [142,143]. In particular, tricyclic antidepressants and mirtazapine treatment induce weight gain increases in TG and LDL levels [144]. Cholesterol elevation and a temporary increase in TGs were also observed after mirtazapine treatment in healthy volunteers compared to the placebo group [145]. Kopf et al. [146] have also demonstrated the increase in TG levels following their amitriptyline therapy. However, these authors considered these changes as a positive therapeutic effect due to the fact that this increase was only observed in patients responding to this therapy.

Despite the previously assumed absence of a pronounced negative effect of selective serotonin reuptake inhibitors on fat metabolism, many studies have shown the opposite. Thus, a number of studies revealed an increase in the level of TC, concentration of TGs, and LDL in the blood of depressed patients following treatment with selective serotonin reuptake inhibitors [143,147,148,149]. Various effects have been shown for different types of antidepressants. For example, Olguner Eker et al. [149] detected an increase in HDL after their escitalopram application, but not following the applications of fluoxetine, sertraline, and venlafaxine. In Beyazyuz’s work, metabolic changes were only observed after the use of paroxetine, citalopram, and escitalopram, but not fluoxetine [148]. Conversely, several studies did not reveal pronounced changes in lipid metabolism following treatment with antidepressants of different classes [150]. The presumed mechanism of the negative effect of selective serotonin reuptake inhibitors on lipid metabolism is the excessive accumulation of TGs in the liver tissues. Thus, it has been shown that fluoxetine injection causes an increase in the levels of lipogenic enzymes and a decrease in the levels of lipolytic enzymes in the livers of mice with modeled depression [143]. Previously, an in vitro experiment also demonstrated that fluoxetine induces lipid accumulation in primary hepatocyte cultures by inhibiting the AMP-activated protein kinase signaling pathway [151].

Along with the negative effects on lipid metabolism, some studies, on the contrary, have revealed a positive role of antidepressants. For example, Hsiao showed that venlafaxine treatment induces a decrease in depressive symptoms accompanied by a decrease in dehydroepiandrosterone, a steroid hormone whose precursor is cholesterol [152]. Venlafaxine treatment in the drug-sensitive patients also caused a decrease in AA levels compared to the drug-resistant group [153]. In addition, Hummel et al. demonstrated that the improvement of patients’ symptoms after the antidepressant treatment was accompanied by an improvement in the LDL/HDL ratio [154].

Several studies have suggested that lipid metabolism itself may play a significant role in response to antidepressant therapy. For example, Sonawalla et al. showed that patients with elevated cholesterol levels may demonstrate lower response to fluoxetine therapy [155]. The authors of another study suggested that baseline-elevated LDL level may increase the binding ability of the serotonin type 1A receptor and thus provide a more pronounced therapeutic effect [156].

Extensive metabolomic studies of depression and antidepressant therapies have identified a number of lipid metabolites that undergo changes with treatment. Analyses of the plasma lipid profile following citalopram and escitalopram administration revealed changes in several types of PC, namely alkyl-PC, lysopPC, and SM (e.g., increases in PC (36:2), PC (30:0), PC (34:3), PC-O (34:2), PC-O (36:3), LPC (24:0), and SM (24:0) and decreases in PC (36:4), PC (38:6), LPC (20:4), and SM (18:1)) [157,158,159]. In view of the fact that phosphatidylcholines are involved in membrane construction and remodeling, the increase in PC may be associated with an increase in the activity of membrane proteins, such as carnitine palmitoyltransferase 1, which is involved in fatty acid beta-oxidation [159]. The increase in the important antioxidant phospholipid PC-O can probably be related to counteracting the oxidative stress that accompanies depressive disorders. Thus, such changes in lipid profiles may indicate a favorable outcome of antidepressant treatments.

However, most studies have not compared patients with diagnosed depression following their treatment and a healthy control group. This makes it difficult to assess the ability of antidepressants to affect lipid metabolism and restore normal lipid profiles [158].

## 4. Conclusions

Lipids represent an extremely significant structural component of the brain, performing various functions related to both the maintenance of cellular function and nerve cell physiology. However, the influence of lipids on the pathologic pathway mechanisms of psychiatric diseases has been undeservedly poorly studied. Disruption of lipid metabolism leads to impaired brain function and the development of neuropsychiatric diseases. Despite the large amount of data concerning the properties of lipids, many of their functions remain poorly understood. In this review, we have demonstrated the role of lipids as potential biomarkers of the most common psychiatric diseases.

We identified a number of studies that observed changes in lipids of various classes during the progression of mental illnesses. Moreover, the altered lipid profile induced by medications, such as antipsychotics and antidepressants, was also described. Lipid markers have the potential to serve as biomarkers for the diagnosis and prognosis of mental illnesses. They can also provide valuable insights into the underlying biological mechanisms of these disorders. In conclusion, while much progress has been made in identifying biological markers of mental illnesses, there is still a need for further research on lipid markers. Understanding the role of lipids in the pathophysiology of mental illnesses could lead to the development of novel diagnostic tools and therapeutic interventions.

## Figures and Tables

**Table 1:** Studies assessing blood lipid alterations in psychiatric disorders at the level of individual lipid species. ↑ increased and ↓ decreased.

**FATTY ACYLS**				
**Type of Lipid**	**Disorder**			
	**SCZ**	**MDD**	**BPD**	**AD**
PUFAs	↑ 8 types [45], ↑ monounsaturated fatty acids and ω-6 PUFAs [80], and ↓ 5 types [40] ↓ 6 types [81]	↓ including eicosapentaenoic acid and arachidonic acid [82], ↑ in plasma, mostly ↓ membrane fatty acids, exp octadectetraenoic, gamma-linolenic acid, and docosadienoic acid [83]. ↑ AA: eicosapentaenoic acid (EPA) ratio [84].	↑ omega-6 PUFA, AA: EPA and AA: EPA+ docosahexaenoic acid (DHA) [84]. ↑ linoleic acid, AA, α-linolenic acid, EPA [85], ↑ ratio of omega-6/omega-3 [86] ↓ DHA 22:6n-3 decreased in membrane [87], and ↓ DHA (22:6n-3) and AA (20:4n-6) [88] ↓ EPA acid [89]. ↓ DHA [85].	↑ n-6:n-3 ratio [90] ↓ N-3 PUFA and ↓ N3:FA ratio in patients with comorbid depressive and anxiety disorder [72]. ↓ DHA in pregnant women [73]. ↓ 3 types of PUFAs in patients with comorbid Parkinson’s disease and AD [65].
SFA	↑ [91], ↑ 4 types [45], ↑ linoleic acid [47], ↓ stearic acid, behenic acid, α-dimorphecolic [50], ↓ lithocholic acid [40], and ↓ 16:0 [92]. Higher levels of total SCFAs, acetic acid, acetic acid/ propionic acid ratio SCZ compared to CTL. The lipid levels were positively associated with acetic acid/propionic acid ratio	↑ azelaic acid, ↓ palmitic acid, dodecanoic acid, and capric acid [82]. ↑ plasma, mostly ↓ membrane exp. palmitic acid [83].	No information.	↓ hexacosanoic acid and 10-oxo-nonadecanoic acid in patients with comorbid Parkinson’s disease and AD [65]. ↑ 3-Hydroxysebacic acid and ↓ 2-Hydroxy-3-methylpentanoic acid [66]. ↑ erythrocyte membrane linoleic acid in patients with anxiety symptoms comorbid to MDD [93].
Carnitine	↑ long chain in FEP [94], ↑ CAR 10:2 [44], ↑ 3 types of carnitine [95], ↓ [34], ↓ 3 types of carnitine [40], ↓ medium and high chain [95], ↓ short-chain CAR in FEP [94], and ↓ 18:2 in FEP [42].	↓ 5 types [59]. ↓ AcCAR [61].	↓ [61].	↓ propionylcarnitine [66].
Transdiagnostic between SCZ, MDD, BPD, and AD: ↓ 10-nitro-9Z,12Z-octadecadienoic acid decreased; ↑ 9,12-octadecadienal, cyclopentaneoctanoic acid, hexadecandioic acid, 12-tridecynoic acid, and caprylic acid [79].				
**SACCHAROLIPIDS**				
**Type of lipid**	**Disorder**			
	**SCZ**	**MDD**	**BPD**	**AD**
MGDG	No information.	↓ [61].	↓ [61].	No information.
**STEROL LIPIDS**				
**Type of lipid**	**Disorder**			
	**SCZ**	**MDD**	**BPD**	**AD**
Cholesteryl esters	↑ in FEP [42]. ↓ CE 16:1 [44].	Plasma tryptophan-kynurenine metabolites and CEs were significantly correlated in the MDD group, but not in the HC group [62]. No difference [96]. ↓ CE [60].	↓ [86]	No information.
Total cholesterol	↓ [52].	↑ in women with depressive symptoms [97]. ↓ in postpartum depression [57].	↑ [98]. ↓ [51,99].	↑ [100]; ↑ in comorbid MDD and GAD [68]. ↓ in alexythimic patents [67], ↓ in patients with comorbid Parkinson’s disease and AD [65], ↓ in the anxious–depressive disorder group [71], and not changed compared to MDD and BPD [101].
Sterols	↑ the ratios of cholestane-3β,5α,6β-triol, 27-hydroxycholesterol, and cholestanol to tchol [55]. ↓ several types of sterol lipids [44].	↑ 7-dihydrocholesterol, ↓ desmosterol, and 14-desmethyl lanosterol in people with depressive symptoms [102].	No information	↓ 11-acetoxy-3β,6α-dihydroxy-9,11-seco-5α-cholest-7-en-9-one in patients with comorbid Parkinson’s disease and AD [67].
Bile acids	↓ [50]. ↓ lithocholic acid [40].	↑ 23-nordeoxycholic acid, ↓ taurolithocholic acid (TLCA), glycolithocholic acid (GLCA), and lithocholic acid (LCA) 3-sulfate ↓ [103]. ↓ chenodeoxycholic acid (CDCA) [74].	No information.	↓ CDCA in highly anxious participants compared to participants with less severe symptoms [74]. ↑ LCA [74].
Calcifediol	↓ [50].	↓ [104]; not changed depressive symptoms in healthy people [78].	↓ [105].	Not changed [78].
↓ 20-oxo-22,23,24,25,26,27-hexanorvitamin D3 [79] transdiagnostic				↓ [79] transdiagnostic. ↓ anxiety symptoms [76,77].
**GLYCEROPHOSPHOLIPIDS**				
**Type of lipid**	**Disorder**			
	**SCZ**	**MDD**	**BPD**	**AD**
PC	↑ 32 types of PC [40], ↓ PC [50], ↓ 46:7 [44], ↓ PC (O-34:2) [40], mostly ↓ membrane PC [31], ↓ PC [45,54,106], PC-O in FEP [107], ↓ PC-P in FEP [42], ↓ PC-O [45,46], ↓ PC-O 38:6 [108], ↓ PUFA-containing PC [53], and ↓ 14 types and ↑ 11 types [34].	↑ [58], ↑ PC 32:1 [59], ↑ PC-O [63] ↑ PC(8:0e/6:0) [34] ↓ 3 types ↑ 5 types [82] ↑ [109] and ↓ PC-O 36:2 [59]	↑ PC [110].	↓ PC O 36:4 (anxiety symptoms) [63] and ↓ LysoPC(0:0/16:0) [66].
	↑ PC-O 16:0-18:1+2O [79] transdiagnostic			
PE	↑ PE 34:2 [40], ↓ [50], ↓ 40:7 [44], ↓ PE (O-34:3), (O-36:6) [40], mostly ↓ membrane PE [31], ↓ PE-P [54,106], ↓ PE-P in FEP [42], ↓ PE-O [46], ↓ PUFA-containing PE [53], and ↓ 2 ↑ 5 [45].	↑ [58], ↑ PE 34:2 36:4 [59], ↓ PE-O [58], ↓ PE-O [59], ↓ PE (16:0/22:6) PE(18:0/22:6) [111], ↑ PE(18:1/0:0), and PE(18:2/0:0) [82].	↓ [61].	No information.
LPC	↑ LPC [40], ↑ LPC 18:1 [44], ↓ [34,43,46] ↓ in FEP [42], mostly ↓ membrane LPC [31] ↑ 4 ↓ 19 [45]	↑ [58,59] ↑ LysoPC (16:0) and LysoPC (18:0) [111], and ↑ LPC [106],	↑ LPC [106].	No information.
Lysophosphatidylethanolamine (LPE)	↑ [34,40] mostly ↓ membrane LPE [31] 3 ↓ 9 ↑ [45]	↑ [58,59]	↑ [61]	↑ lysoPE(18:2(9Z,12Z)/0:0) [66].
PS	↑ LPS 21:0 [44], ↓ 43:2 [44], ↓ 10, ↑ 13 membrane PS [31],	↓ [61]	↓ [61]	No information.
↑ DGTS 16:0/18:1 [79] transdiagnostic				
**SPHINGOLIPIDS**				
**Type of lipid**	**Disorder**			
	**SCZ**	**MDD**	**BPD**	**AD**
Sphingomyelin	↑ SM with PUFA (C18:1 and C24:1), ↓ 12 types [44] mostly ↓ SMs with SFA (C16:0, C20:0, and C24:0) [45], and mostly ↓ membrane SM [31]	↑ [63], ↓ PhSM [61], and ↓SM 39:1 [58].	↓ SM and phSM [61].	↓ SPM 23:1 (anxiety symptoms) [63].
Ceramide	↑ Cer (d18: 1/16: 0), Cer (d18: 1/18: 0) и Cer (d18: 1/24: 1) [112], ↓ 44:1 [44] ↓ 22, ↑ 20 membrane Cer [31]	↑ Cer elevated [113], ↑ Cer and HexCer [114] ↓ CerG2GNAc1(d38:4) [34], ↓ CerG2GNAc1 [61]	↑ Cer and HexCer elevated [114] and ↑ Cer22:0 [115]	↑ Cer 20:0 in Parkinson’s disease patients with anxiety symptoms [75].
Ganglioside	No information	↑ monosialotetrahexosylganglioside 2 (GM2) [61]	↑ GM2 [61].	No information.
Other	↓ C16 sphinganine [50]; ↓ glycosphingolipids [44].		↑ total sphingolipids [68].	↓ N-(hexadecanoyl)-deoxysphing-4-enine-1-sulfonate in patients with comorbid Parkinson’s disease and AD [65].
**GLYCEROLIPIDS**				
**Type of lipid**	**Disorders**			
	**SCZ**	**MDD**	**BPD**	**AD**
TG	↑ 20 types [41], ↑ [43,52,116] ↑ in FEP [42], ↑ membrane TG [31], and ↓ 3 types [44].	↑ [58,59] and ↓ TG [60]. ↑ [34].	↑ [51,61,114]. ↓ TG [99].	↑ in alexythimic patents [67], ↑ comorbid MDD with anxiety [68,69,71], ↑ anxiety symptoms [70], and ↓ in patients with comorbid Parkinson’s disease and AD [65].
DG	↑ membrane [31].	Not changed [60].	↑ [114].	↓ 6 types in patients with comorbid Parkinson’s disease and AD [65]
**PRODUCTS OF LIPID METABOLISM**				
**Type of lipid**	**Disorder**			
	**SCZ**	**MDD**	**BPD**	**AD**
Coenzyme Q10	No information.	↓ [117].	↓ [61].	No information.
Malondialdehyde	↑ [49,118,119,120]	↑ [121,122].	↑ [123].	↑ [124]; ↑ post-stroke anxiety [64].
Lipoproteins	↓ HDL [52]; ↓ HDL, LDL, and ApoE [51].	↑ LDL [34], ↓ HDL [125], ↓ HDL-c postpartum [57], and ↓ HDL-C [62].	HDL ↓, LDL, and apolipoprotein E (ApoE) ↓ [51].	↑ LDL [67,68], ↓ LDL in patients with comorbid Parkinson’s disease and AD [65], ↓ LDL (tension-anxiety symptoms) [126], ↓ HDL [67,68,69,127,128], ↑ HDL (tension–anxiety symptoms) [126], ↑ VLDL [67], ↑ LDL/HDL [67], ↑ TC/HDL [67,128], ↑ ApoB [71], ↓ ApoB in patients with comorbid Parkinson’s disease and AD [65], and ↑ ApoA [71].

## Data Availability

Data sharing not applicable No new data were created or analyzed in this study.

## Supplementary Materials

The following supporting information can be downloaded at: https://www.mdpi.com/article/10.3390/metabo14020080/s1, Table S1: Studies assessing blood lipid alterations in psychiatric disorders at the level of individual lipid species.

## Abbreviations

AA	Arachidonic acid
AD	Anxiety disorder
Apo	Apolipoprotein
BPD	Bipolar disorder
CAR	Acylcarnitine
CDCA	Chenodeoxycholic acid
CE	Cholesteryl ester
Cer	Ceramide
CNS	Central nervous system
DG	Diacylglycerol
DHA	Docosahexaenoic acid
FEP	First-episode psychosis
GAD	General anxiety disorder
GLCA	Glycolithocholic acid
GM	Monosialotetrahexosylganglioside
GP	Glycerophospholipid
HDL	High-density lipoprotein
HPA	Hypothalamic–pituitary–adrenal axis
IP3	Inositol triphosphate
LCA	Lithocholic acid
LDL	Low-density lipoprotein
LPC	Lysophosphatidylcholine
LPC-O	Lysoplasmanyl-phosphatidylcholine
LPC-P	Lysoplasmenyl-phosphatidylcholine
LPE	Lysophosphatidylethanolamine
LPI	Lysophosphatidylinositol
MDD	Major depressive disorder
MOCA	Montreal Cognitive Assessment
PANSS	Positive and Negative Syndrome Scale
PC	Phosphatidylcholine
PC-O	Octadecyl-phosphatidylcholine
PC-P	Plasmenyl-phosphatidylcholine
PE	Phosphatidylethanolamine
PE-O	Octadecyl-phosphatidylethanolamine
PE-P	Plasmenyl-phosphatidylethanolamine
PG	Prostaglandin
PI	Phosphatidylinositol
PIP	Phosphatidylinositol phosphate
PLA2	Phospholipase A2
PS	Phosphatidylserine
PUFAs	Polyunsaturated fatty acids
RBANS	Repeated Battery for the Assessment of Neuropsychological Status
SCZ	Schizophrenia
SFA	Saturated fatty acid
SM	Sphingomyelin
SP	Sphingolipid
SREBP	Sterol regulatory element-binding protein
TAG	Triacylglycerol
TC	Total cholesterol
TG	Triacylglycerol
TGH	Triacylglycerol hydrolase
TLCA	Taurolithocholic acid
UHR	Ultra-high risk
VLDL	Very low-density lipoprotein
