# Supplementary material for: Lipids in Psychiatric Disorders: Functional and Potential Diagnostic Role as Blood Biomarkers
Source: Metabolites. 2024 Jan 23;14(2):80. doi: 10.3390/metabo14020080 (PMC10890164; doi:10.3390/metabo14020080)
Supplement: Supplementary file 1 [file metabolites-14-00080-s001.zip › metabolites-2737554-supplementary.pdf]

Supplementary Materials

Table S1. Studies assessing blood lipid alterations in psychiatric disorders at the level of individual lipid species.

	Study	Disease	Medication Status	Sample Size	Lipid Classes	Main Findings
[91]	Li 2022	SCZ	medicated	SCZ=44, CTL=35	SCFAs	Higher levels of total SCFAs, acetic acid, acetic acid/ propionic acid ratio SCZ compared to CTL. The lipid levels were positively associated with acetic acid/ propionic acid ratio.
[41]	Tkachev 2021	SCZ	pre- and post- treatment	SCZ=92	322 different lipids	20 triglyceride species increased in individuals with the least improvement in PANSS scores, but not in those with the largest reduction in PANSS scores.
[40]	Liu 2021	SCZ	medicated	SCZ=76, CTL=50.	LPE, PE, PC, LPC, PUFAs	LPEs (LPE 16:1 sn1, LPE 16:1 sn2, and LPE 22:5 sn2) and LPC 16:0 sn2 were higher, CAR (8:1, 10:1, 10:2) and PUFA 16:2, 18:2, 18:3, 20:4, 22:6) were lower in SCZ drug-naïve patients. Lithocholic acid were lower in SCZ patients. PE (O-34:3) and the PE (O-36:6) decreased, but the PE 34:2 increased.
[52]	Nandeesha 2023	SCZ	medicated	SCZ=200, CTL=169.	TC, TG, HDL, LDL, VLDL	TG increased and HDL-C was reduced in SCZ. TC and TG was negatively correlated with cognitive score.
[50]	Song 2023	SCZ	pre- and post- treatment vs control	SCZ=43, CTL=29	PC,PE, C16 Sphinganine, PUFAs	Lower levels of LysoPC, PC, PE, C16 sphinganine, sulfate, glycocholic acid, taurocholic acid, stearic acid, behenic acid, α-dimorphecolic, Calcifediol, L- hydroxyproline in SCZ compared to CTL. After medication with risperidone or olanzapine, the levels of LysoPC, PC, PE, C16 sphinganine and adrenic acid were significantly increased, while linoleic acid, oleic acid, palmitoleic acid, γ-linolenic acid, oxoglutaric acid were significantly decreased.

[55]	Guidara 2022	SCZ	medicated	SCZ=40, CTL=40	oxysterols	The ratios of cholestane-3β,5α,6β-triol, 27-hydroxycholesterol (27-OHC) and Cholestanol to Tchol increased SCZ compared to CTL. For the SCZ patients, the plasma 24- hydroxycholesterol levels were positively correlated with PANSS but negatively correlated with the MoCA. The ratio Cholestanol to Tchol was negatively correlated with MOCA scores and positively correlated with PANSS general.
[34]	Wang 2022	SCZ	medicated	SCZ=31, CTL=32	30 lipid classes and 782 lipid species	Levels of acylcarnitine and PE were lower, whereas those of LPC, LPE, LPI, PIP, and PIP2 were higher in the SCZ compared to CTL
[44]	Costa 2023	SCZ	medicated	SCZ=30, CTL=30	LPC, LPS, LPE, CE, Cer, CAR, SM, PE, TG, PC, PS	Increased: LPS 21:0, LPC18:1, CAR 10:2 Decreased: CE 16:1, SM 12 types, Cer 44:1, PE 40:7, TG 51:7, 53:7, 54:7, PC 46:7, PS 43:2
[31]	Li 2022	SCZ	medicated	SCZ=80, CTL=40	812 different lipids	244 lipids were significantly reduced (22 types of CER, 25 LPC, 12 LPE, 50 PC, 103 PE, 6PI, 10 PS, 13 SM), and 143 increased (20 types of CER, 32 TG, 4 DG, 3 LPC, 3 LPE, 17PC, 35 PE, 7PI, 13 PS, 5 SM)
[47]	Frajerman 2023	SCZ	medicated one-year follow-up	32 converters (C) and 29 non-converters (NC) persons at ultra-high risk of psychosis (UHR)	Fatty acids, phospholipids and sterols	Among UHR individuals, elevated baseline membrane linoleic acid level was associated with conversion to psychosis. Combining sterols, fatty acids, and membrane composition improved the prediction of psychosis onset.
[56]	Zhang 2023	SCZ	medicated	SCZ=105, CTL=105	TC	TC levels were positively associated with RBANS subscale scores of immediate memory and language.
[95]	Cao 2019	SCZ	pre- and post- treatment vs control	SCZ=225, CTL=175	CAR	Medium and high chain CAR decreased in pre-treatment SCZ compared to CTL, even lower post-treatment SCZ.
[106]	Wood 2015	SCZ	medicated	SCZ=23, CTL=27	LPE-P, LPC-P, PE-P, PC-P	PE-P, PC-P decreased in SCZ compared to CTL

[94]	Kriisa 2017	SCZ	pre- and post- treatment vs control	SCZ=38, CTL=37	CAR	Long-chain CAR increased in FEP compared to CTL; short-chain CAR decreased in FEP compared to CTL; CAR decreased in FEP after medication
[107]	Leppik 2020	SCZ	pre- and post- treatment vs control	SCZ=38, CTL=37	PC, PC-O, SM	PC, PC-O reduced in FEP compared to CTL; no difference in medicated SCZ and CTL
[42]	Yan 2018	SCZ	pre- treatment vs control and pre- vs post- treatment	SCZ=20, CTL=29	445 lipids belonging to 17 lipid TG, CE, LPC, PC-P, PE-P, CAR	TG, CE increased in FEP compared to CTL; LPC, PC-P, PE-P decreased in FEP compared to CTL; CAR 18:2 decreased in FEP compared to CTL
[46]	Wang 2019	SCZ	medicated and non-medicated	SCZ=119 (28 FEP), CTL=109	PC, PE, LPC, LPE, SM, PC-O, PE-O	LPC, PC-O, PE-O decreased in SCZ compared to CTL; SM increased in SCZ compared to CTL; PC both increased and decreased depending on lipid species; no difference FEP vs recurrent SCZ
[92]	Cao 2020	SCZ	recently non-medicated (1 month)	SCZ=113, CTL=111	Car, LPC, LPC-O	LPC decreased in SCZ vs CTL; CAR increased 10:1, 18:1, 16:0
[108]	He 2012	SCZ	medicated and non-medicated	SCZ=216, CTL=265	PC-O 38:6	PC-O 38:6 decreased in SCZ compared to CTL
[43]	Orešič 2012	SCZ	medicated	SCZ=19, CTL=53; twins	LPC, PC, PE, PG, PS, PA, Cer, MAG, DAG, TAG	Twin study. TG increased in SCZ compared to CTL; LPC lipid species decreased in SCZ compared to CTL. difference between SCZ and CTL not included in table form
[53]	McEvoy 2013	SCZ	medicated and non-medicated	SCZ=20, FEP=20, CTL=29	PC, PE	Decreased PUFA-containing PC and PE lipid species in SCZ compared to CTL. Lipid species containing particular fatty acids, no bulk structure
[116]	Solberg 2016	SCZ	medicated	SCZ=55	TG, membrane PUFAs (blood cells)	Increased TG in SCZ compared to CTL. No individual TG species; esterified membrane (blood cells) PUFAs
[54]	Kaddurah-Daouk 2012	SCZ	recently non-medicated and drug naïve	SCZ=20+20 (naive and recently unmedicated), CTL=17	PC-P, PE-P	PC-P, PE-P decreased in both drug-naïve and medicated SCZ compared to CTL; lipid species containing particular fatty acids, no bulk structure

[45]	Wang 2021	SCZ	28 patients drug-naïve, the other patients have recurrent schizophrenia (91) who had not taken any antipsychotic drugs that may modify lipid metabolism for at least one month	SCZ=119, CTL=109	110, including 16 FFAs, 25 phosphatidylcholines, 23 lysophosphatidylcholines, 11 phosphatidylcholine plasmalogens, 7 phosphatidylethanolamines, 9 lysophosphatidylethanolamines, 6 phosphatidylethanolamine plasmalogens, and 13 sphingomyelins	SFA increased: 14:0, 16:0, 17:0, 20:0,<br>PUFA increased: 16:1, 17:1, 20:1, 20:2, 22:1, 22:2, 22:4, 24:1<br>LPC: 4 increased 19 decreased<br>PC: 14 types decreased 11 types increased<br>PC-O decreased<br>LPE 3 decreased 9 increased<br>PE 2 decreased 5 increased<br>PC-P decreased<br>SM: 6 increased, 7 decreased. Elevated SMs were associated with increased breakdown of PCs, SMs with unsaturated fatty acid residues (C18:1 and C24:1) were higher, most SMs containing saturated fatty acid residues (C16:0, C20:0, and C24:0) were lower<br>Increased SMs were associated with increasing breakdown of PCs
[81]	Zhou 2020	SCZ	drug-naïve	first-episode SCZ=40, CTL=52	PUFAs, SFAs	Significant reductions of 16:0, 18:1n9c 18:2n6c, and 20:4n6 20:4n6 22:6n3 levels in SCZ compared to CTL. Disrupted metabolism of fatty acids especially in saturated and n-6 fatty acid families were observed by comparing correlations between precursor and product fatty acid levels within each fatty acid family.
[80]	Yang 2017	SCZ	Medicated	SCZ=110, CTL=109	PUFAs	Monounsaturated fatty acids and ω-6 PUFAs were significantly increased in SCZs compared with CTL. Desaturation from saturated fatty acids to Monounsaturated fatty acids and β-oxidation were enhanced, as estimated by the ratios of products to precursors.
[112]	Tkachev 2021	SCZ	Medicated	SCZ=82, CTL=138	Cer	Cer (d18: 1/16: 0), Cer (d18: 1/18: 0) и Cer (d18: 1/24: 1). Among these compounds, Cer (d18: 1/18: 0) showed the strongest largest difference between patients and controls.

[118]	Hurşitoğlu 2021	SCZ	Medicated	SCZ=30, CTL=30	malondialdehyde	Significantly increased. No significant correlation was found (p>0.05) between malondialdehyde, superoxide dismutase and catalase activity and PANSS scores or the chlorpromazine equivalent and clinical characteristics.
[119]	Güneş 2016	SCZ	Medicated	SCZ=41, CTL=43 (11 taking typical antipsychotics, 19 taking atypical antipsychotics, 11 taking combined antipsychotics).	malondialdehyde	Significantly increased, depend on treatment
[120]	Uddin 2021	SCZ	Medicated	SCZ=63, CTL=63	malondialdehyde	Increased
[51]	Wei 2022	SCZ	Medicated	SCZ=13329, CTL=5810	TG, TC, VLDL, VDL,LDL, APOB	TG were higher in SCZ and BPD groups, and levels of cholesterol, HDL, LDL, and ApoB were lower in SCZ and BPD groups.
[34]	Wang 2022	MDD, SCZ	Medicated	MDD = 35, SCZ = 31, CTL=32	TG, PI, PC, Cer, lipoproteins	PI(16:1) decreased in the MDD group. TG(18:3/18:2/18:3), PC(8:0e/6:0), CerG2GNAc1(d38:4), LDL increased in MDD.
[113]	Gracia-Garcia 2011	MDD	Medicated	MDD=23, CTL=23	Cer	Cer elevated in MDD compared to CTL
[58]	Liu 2016	MDD	medicated and non-medicated	MDD=60, CTL=59+ MDD=75 and CTL=52	LPC, LPE, PC, PE, PI, TG	LPC, LPE, PC, PE, PI, TG increased with increase of symptome severity; PE-O decreased with increase of symptom severity (HAMD) SM 39:1, 2 significantly decreased in MDD subjects, while LPE 20:4, PC 34:1, PI 40:4, and TG 44:2 increased
[59]	Liu 2015	MDD	medicated and non-medicated	MDD=60, CTL=59 for validation MDD=75 and CTL=52	LPC, LPE, PC, PE, PI, TG, CAR	LPC 16:1 sn-1 16:1 sn-2, LPE 16:0 sn-1, 16:0 sn-2 18:1 sn-2 22:5 sn-1 16:1 sn-1, PC 32:1, PE 34:2 36:4, TG increased in MDD compared to CTL; PC-O 36:2, PE-O 34:3 36:5 38:7 36:2, 5 types of CAR decreased in MDD compared to CTL
[63]	Demirkan 2013	MDD	Medicated	samples=742, family study	SM, PC	SM and PC-O significantly associated with symptome severity (HADS-D)

[60]	Kim 2018	MDD	recently non-medicated (8 weeks)	MDD=20, CTL=10 + 25*3 validation	TG, DG, PS, PI, CE	TG mostly decreased in MDD compared to CTL;<br>DG not changed, CE increased, PI decreased. Results not reproduced in validation, study not used for comparison
[111]	Cai 2019	MDD	Drug naïve	MDD=30, CTL=30	PE, LPC	LysoPC (16:0) LysoPC (18:0) increased<br>PE (16:0/22:6) PE (18:0/22:6) decreased in MDD compared to CTL.
[61]	Zhang 2022	MDD, BPD	Medicated	MDD = 28, BDD = 22, CTL=25	different lipids	Levels of MGDG, phSM, CerG2GNAc1, AcCa and PS were decreased while GM2 and PIP were increased in MDD when compared with CTL. Levels of MGDG, CoQ10, SM, phSM, AcCa, PS, and PE were decreased while GD2, GM2, TG, LPE, PIP, and PIP2 were increased in BPD when compared with CTL. Levels of LPE and LPI were decreased while PE were increased in MDD when compared with BPD.
[79]	Tao 2022	MDD, BPD, SCZ transdiagnostic subtypes	medicated and non-medicated	MDD = 105, BPD = 132,<br>SCZ = 112,<br>CTL=198	9,12-Octadecadienal, 10-nitro-9Z,12Z-octadecadienoic acid, Cyclopentaneoctanoic acid, Hexadecandioic acid,12-Tridecynoic acid,Caprylic acid, 20-oxo-22,23,24,25,26,27-hexanorvitamin D3, 4-amino-3-methylbutanoic acid, DGTS 16:0/18:1	The 10 identified lipids could help identify homogeneous transdiagnostic subtypes across psychiatric disorders.
[82]	Zhou 2019	MDD From 6 to 18 years	52 drug naive, 32 with treatment	MDD=84<br>CNL=50	PUFAs, SFA, PC, PE	alpha-Linolenic acid, Arachidonic Acid, cis-9-Palmitoleic acid, Eicosapentaenoic acid, Linoleic acid, Oleic acid, decreased. Azelaic acid increased, Palmitic acid Dodecanoic acid,Capric acid (SFA) decreased<br>PC(10:0/14:1), PC(12:0/22:5), PC(20:5/24:4) decreased

						PC(13:0/22:2) PC(18:0/0:0) PC(18:1/0:0) PC(18:2/24:4) PC(20:5/0:0) increased PE(18:1/0:0) PE(18:2/0:0) increased
[83]	Assies 2010	recurrent MDD	Medicated	MDD=137 CNL=65	PUFAs, SFA	Plasma fatty acids increased, membrane fatty acids mostly decreased. Plasma: Linolenic acid, Linoleic acid, Gamma-linolenic acid, Docosadienoic acid, Myristoleic acid, Palmitoleic acid, Vaccenic acid, Hypogeic acid, Oleic acid, Erucid acid, Nervonic acid. Sfa: Myristic acid, Palmitic acid, Arachidic acid, Behenic acid Erythrocyte: Octadectetraenoic acid (increased), Docosapentaenoic acid, Docosahexaenoic acid, Gamma-linolenic acid(increased), Arachidonic acid, Docosatetraenoic acid Docosapentaenoic acid Docosadienoic acid Myristoleic acid Palmitoleic acid Hypogeic acid Nervonic acid. SFA: Palmitic acid (increased) Arachidic acid Behenic acid Lignoceric acid
[62]	Kuwano 2018	MDD	Drug naïve	MDD=15 CNL=19	TC, HDL-C, LDL-C, 62 plasma lipid species including LPC, LPE, PC, PE, cholesteryl ester (CE), TG	Plasma tryptophan-kynurenine metabolites and cholesteryl esters were significantly correlated in MDD group, but not in HC group. HDL-C correlated negatively with depressive symptoms.
[103]	Sun 2022	MDD	Drug naïve	MDD=31 CNL=29	bile acid	Increased 23-nordeoxycholic acid decreased taurolithocholic acid, glycolithocholic acid, and lithocholic acid 3-sulfate in MDD compared to CTL
[74]	MahmoudianDehkordi 2022	MDD, Anxiety symptoms	Drug naïve	MDD=208	bile acid	Chenodeoxycholic acid was significantly lower at baseline in both severely depressed and highly anxious participants compared to participants with less severe symptoms. Lithocholic acid was higher in the more anxious participants.

[104]	Köhnke 2022	MDD	Medicated	MDD=639 CNL=530	Calcif	lower 25(OH)D levels than CTL. Patients with atypical depression had the lowest levels. Symptom severity was inversely related to 25(OH)D.
[109]	Stirton 2021	MDD	Medicated	MDD=48 CNL=9	total OxPCs	Increased compared to CTL
[117]	Maes 2009	MDD	Medicated	MDD=35 CNL=22	CoQ10	CoQ10 was significantly lower in depressed patients compared to CTL. significantly lower in patients with treatment resistant.
[124]	Islam 2018	MDD	Medicated	MDD=247 CNL=248	Malondialdehyde	Malondialdehyde levels were higher in patients compared to CTL
[122]	Alvarez-Mon 2022	MDD	Medicated	MDD=30 CNL=20	Malondialdehyde	Malondialdehyde levels were higher in patients compared to CTL
[125]	Uyar 2022	MDD	Different medication status	MDD=20 CNL=124	Lipoproteins	HDL levels were lower in the MDD patients who had never received treatment in comparison to those who had received treatment.
[57]	Pillai 2018	Post-partum depression		MDD=186 CNL=250	Lipoproteins, TC	Low serum levels of TC and HDL-c were significantly low in postpartum depression women with severe depressive symptoms.
[96]	Mamalakis 2006	Depressive symptoms In health people	No medication	150 males	CE	There were no significant differences.
[97]	Beydoun 2015	Depressive symptoms In health people	baseline depressive symptoms as predictors for longitudinal change in lipid profile	Men: n=826 ; Women: n=1099	TC	A higher level TC was linked to faster increase in depressive symptom scores, only among women.
[102]	Cenik 2017	Depressive symptoms In health people	No medication	3117 adults	sterols	Increased 7-Dehydrocholesterol, decreased desmosterol, 14-desmethyl lanosterol in people with depressive symptoms
[78]	Casseb 2019	Depressive symptoms and	No medication	36 adults	Calcifediol	No association with depressive and anxiety symptoms

		anxiety symptoms In health people				
[87]	McNamara 2015	BPD	Medicated	BD=40, CTL=40	PUFAs	Docosahexaenoic acid (DHA, 22:6*n*-3), EPA+DHA (Omega-3 Index) decreased in erythrocyte membrane
[86]	Sobczak 2004	BPD	Drug naïve	First-degree relatives of patients with BD=30, CTL=15	PUFAs	Increased ratio of omega-6/omega-3 fatty acids in serum
[99]	Huang 2018	BPD	medicated	manic BD=32, CTL=64	TG, TC	TG increased in serum, TC decreased
[105]	Boerman 2016	BPD	medicated	BD=118	Calcifediol	Calcifediol decreased in blood
[98]	Ribeiro 2017	BPD	medicated	BD=14, CTL=21	sphingolipids glycerophospholipids	Sphingolipids, phosphoinositols increased in serum
[115]	Brunkhorst-Kanaan 2021	BPD	medicated	BD=6, CTL=98	Cer	Cer22:0 increased in plasma
[51]	Wei 2022	BPD	medicated	BD=4061, CTL=5810	TG, TC	TG increased, TC decreased in blood
[110]	Costa 2019	BPD, SCZ	Drug naïve	BPD=27, SCZ =28, CTL=30	LPC, PC, SM, Cer, LPE, PE, TG, PI, LPS, PS, CAR	LPC, PC increased in BPD and SCZ vs. CTL
[123]	Aydemir 2014	BPD	medicated	BPD=51, CTL=50	malondialdehyde	malondialdehyde higher in BPD compared to CTL
[114]	Brunkhorst-Kanaan 2019	BPD, MDD	medicated	BPD=32, CTL=405	Cer, HexCer, DG	Cer and HexCer elevated in BPD compared to CTL, DG increased
[84]	Scola 2018	BPD, MDD	medicated	BPD=87, MDD=34, CTL=31	PUFAs	higher levels AA: EPA and AA: EPA+DHA in patients with BPD. Omega-6 present a positive correlation with illness duration in patients with BPD and AA: EPA ratio positively associated with illness duration in MDD group.
[89]	Saunders 2015	BPD	medicated and non-medicated	BPD=27, CTL=31	PUFAs	Eicosapentaenoic acid decreased in BPD vs CTL
[85]	Pomponi 2013	BPD	medicated	BPD=42, CTL=57	PUFAs	Increased Linoleic acid Arachidonic acid α-Linolenic acid EPA, decreased DHA

[90]	Mongan 2021	GAD	unknown	GAD=386<br>CTL=3571	PUFAs	Positive association of n-6:n-3 ration with GAD at age 24
[67]	De Berardis 2015	GAD	drug-naïve	GAD=70	HDL-C, LDL-C, total cholesterol (TC), TG, VLDL-C, TC/HDL-C ratio, LDL-C/HDL-C ratio	Alexithymic patients (44,3%) showed higher TG, VLDL-C, LDL-C, LDL-C/HDL-C, TC/HDL-C ratio and lower TC and HDL-C
[101]	Lacerda 2000	GAD	free of medication	GAD=23<br>PD (panic disorder)=41<br>MDD=21	TC	No significant differences in cholesterol level between GAD, PD and MDD
[100]	Peter 2002	GAD	medicated	GAD=60<br>OCD=60<br>CTL=60	TC	Patients with GAD and OCB have elevated cholesterol level compared to CTL
[68]	Sevincok 2001	GAD, MDD, Comorbid GAD and MDD	Drug-free for at least 1 month	GAD-MD=40<br>MDD=27<br>GAD=26<br>CTL=24	TC, TG, HDL-C, LDL-C	Comorbid MDD and GAD is characterized by higher total cholesterol, TG, higher LDL-C and lower LDL-C
[69]	Wang 2016	Depression with anxiety	free of medication or recently non-medicated (2 month)	Depression with anxiety=29<br>Depression without anxiety=31	TC, TG, HDL-C, LDL=C, lipoprotein A	TG levels statistically higher in depression with anxiety group. Negative correlation between HDL levels and anxiety score
[127]	Veen 2009	MDD and/or AD	Recently non-medicated with exception of a low dose of benzodiazepine	Patents with depression and/or anxiety disorder=72<br>CTL=42	TC, TG, LDL, HDL	Elevated basal cortisol concentration were independently associated with a less favorable lipoprotein profile in patients. Patients showed lower HDL compared to CTL
[93]	Jadoon 2012	Anxiety symptoms in older patients with MDD	medicated	N=132	PUFAs	Erythrocyte membrane linoleic acid levels had a curvilinear association with anxiety symptoms
[72]	Thesing 2018	AD	medicated	Current pure depressive disorder=304<br>Current pure anxiety disorder=548	PUFAs	Comorbid depressive and anxiety disorder patients had lower N-3 PUFA and lower N3:FA ratio

				Current comorbid anxiety and depression disorder=529<br>Remitted anxiety/depressive disorder=897<br>HC=634		
[63]	Demirkan 2013	Anxiety simptoms	medicated	Population study (Dutch-family)<br>N=742	148 different lipids (phospho- and sphingolipids)	The inverse correlation between PC O 36:4, SPM 23:1 and anxiety symptoms
[128]	Huang 2003	Anxious state	unknown	Anxious state=20<br>Depression state=103<br>CTL=39	TC, TG, HDL-C, VLDL, LDL-C, TC/HDL ratio, HDL/LDL ratio	Significant differences in HDL and TC/HDL among patients with anxious state, depression state and CTL in men
[73]	Verly-Miguel 2015	AD during pregnancy	drug-naïve	Cross-sectional study<br>N=228 pregnant women	PUFAs	Serum concentration of DHA was inversely associated with the occurrence of anxiety disorder during early pregnancy
[76]	Huang 2014	Anxiety symptoms	Not medicated	Population study: 498 pregnant women with anxiety symptoms measured by Stress Scales 21-item Short Form (DASS-21)	Calcifediol	A 1 ng/mL lower calcifediol was associated with 0.043 higher DASS-21 Anxiety Scores
[77]	Chen 2020	Anxiety symptoms	Not medicated	Population study: 327 young adults	Calcifediol	higher serum calcifediol concentrations were significantly related to lower scores on the State-Trait Anxiety Inventory
[65]	Dong 2021	AD associated with Parkinson disease	unknown	PDA (with anxiety)=35<br>PDNA (no anxiety)=35	TC, TG, HDL-C, LDL-C, Apo-A1, Apo-B, lipoprotein a, metabolomics study of different lipids	PDA patients showed lower levels of TC, TG, LDL-C, apolipoprotein B compared to the healthy control and PDNA patients.

				HC=35		14 types of lipids determined by metabolome analysis were decreased in PDA group compared to PDNA and HC: 1 sterol lipid (11-acetoxy-3β,6α-dihydroxy-9,11-seco-5α-cholest-7-en-9-one), 6 glycerolipids (DG(13:0/18:2(9Z,12Z)/0:0)[iso2], DG(14:0/18:1(11Z)/0:0), DG(15:0/18:1(11Z)/0:0), DG(15:0/16:1(9Z)/0:0)), (DG(14:0/18:2(9Z,12Z)/0:0), DG(15:0/18:2(9Z,12Z)/0:0), 5 fatty acyls (Hexacosanoic acid, 15-tetracosenal, 2R-HpOTrE, 13-OxoODE, 10-oxo-nonadecanoic acid), 1 sphingolipid (N-(hexadecanoyl)-deoxysphing-4-enine-1-sulfonate), 1 prenol lipid (7,8-Didehydroastaxanthin)
[71]	Pistorio 2011	Anxiety disorder	medicated	AD group=28<br>Depression group=39<br>Anxious-depressive disorder group=13<br>Eating disorders group=15<br>CTL=60	TC, TG and apoA and B	Lower level of cholesterol in the Anxious-depressive disorder group compared to other groups. Higher triglycerides level in AD group and anxious-depressive disorder group compared to other groups. Higher level of Apolipoprotein A in the anxious-depressive group, and higher level of Apolipoprotein B in the AD group compared to other groups
[126]	Lieberman 2012	Tension-Anxiety symptoms	Not medicated	Female volunteers from recruits at the USMC training facility, n=35	FFA, LDL, HDL, TG, dehydroepiandrosterone-sulfate (DHEA-S)	Increases HDL, FFA, DHEA-S were associated with tension-anxiety. LDL was negatively correlated with tension-anxiety
[66]	Kui 2022	Anxiety disorder	medicated	AD=18<br>CTL=31	Metabolomics study	Higher level of 3-Hydroxysebacic acid, LysoPE(18:2(9Z,12Z)/0:0) in AD group compared to CTL,<br>Lower level of Propionylcarnitine, LysoPC(0:0/16:0), 2-Hydroxy-3-methylpentanoic acid in AD group compared to CTL

[70]	Hu 2018	Anxiety symptoms	Not medicated	1754 participants, who completed the STAI-T	TG, HDL	higher STAI-T scores were associated with higher triglyceride levels
[75]	Xing 2016	Anxiety symptoms in PD patients	unknown	PD patients with cognitive impairment=38<br>PD patients with no cognitive impairment=40<br>CTL=40	Cer	Cer C 20:0 was positively associated with anxiety symptoms in patients with Parkinson disease and cognitive impaiment
[124]	Islam 2013	GAD	medicated	GAD=50<br>CTL=51	Malondialdehyde	Serum malondialdehyde content was significantly higher in GAD patients compared to the controls
[64]	Liu 2018	Post-stroke anxiety	medicated	Anxiety group=49<br>Non-anxiety group=154	Malondialdehyde	Malondialdehyde level was increased in patients with post-stroke anxiety compared to post-stroke patients without anxiety
